# Somatostatin Decorated Quantum Dots for Targeting of Somatostatin Receptors

**Published:** 2018

**Authors:** Ahmed Abdelfattah Hafez Abdellatif, Wael Abdellah Abdelhafez, Hatem Abdelmunsef Sarhan

**Affiliations:** a *Department of Pharmaceutics and Industrial pharmacy, Faculty of Pharmacy, Al-Azhar University, Assuit, Egypt. *; b *Department of Pharmaceutics and Industrial pharmacy, Faculty of Pharmacy, El-Minia University, El-Minia, Egypt.*

**Keywords:** Quantum dots, Somatostatin receptors, Somatostatin, Cellular uptake, Receptor targeting

## Abstract

Due to the unique optical properties like high brightness and narrow emission bands of Quantum dots, it is used as simple fluorescence materials in bio-imaging, immunoassays, microarrays, and other applications. To easy invistigate cell lines that overexpressed somtostatin receptors, somatostatin (SST) was conjugated with Quantum dots carrying PEG amine (Qdots-PEG-NH_2_). The conjugation of SST to Qdots-PEG-NH_2_ started with the thiolation of SST using Traut’s reagent. Moreover, the Qdots-PEG-NH_2_ were subsequently activated by 500-fold molar excess of sulfosuccinimidyl 4-(N-maleimidomethyl) cyclohexane-1-carboxylate (sulfo-SMCC) dissolved in phosphate buffer. The Qdots-PEG-NH_2_-sulfo-SMCC was conjugated to the thiolated-SST to form Qdots-SST. The number of sulfhydryl groups can be controlled by the molar ratio of Traut´s reagent to SST. Thiolation was necessary for the conjugation of SST to Qdots-PEG-NH_2_. This was achieved by reacting the SST with Traut’s reagent in a 1:1 molar ratio. Ellman’s reagent was used to determine the number of sulfhydryle groups. Furthermore, cellular uptake study on triple negative breast cancer cells (HCC-1806) showed that the numbers of Qdots-SST per cell were signiﬁcantly higher compared to unmodified Qdots-PEG-NH_2_ when quantified using inductively coupled plasma optical emission spectroscopy (ICP-OES). Moreover, the binding of Qdots-SST to cells can be suppressed by addition of free SST, indicating that the binding of Qdots-SST to cells is due to receptor-specific binding.

## Introduction

Quantum dots (Qdots) are semiconductor nanoparticles, with diameters from 1 to 10 nanometers. Qdots have attracted tremendous interest due to their unique optical properties (1-3). Qdots have a higher molar extinction coefficient compared to organic dyes, making them brighter in photon-limited *in-vivo* studies, which means that they can absorb light efficiently (4). Furthermore, Qdots are highly photostable compared to other fluorophores making them easier to be detected by fluorescence microscopy (5). Qdots are size-tunable and emit light with different wavelengths depending on their size. Larger particles emit light at the red end of the visible spectrum, while smaller particles emit at shorter wavelength (6, 7). A great advantage of Qdots over organic fluorophores for *in-vivo* applications is that they are resistant against photobleaching (8), and also they have long time blood circulation times and are stable in the blood circulation for several months (9). Nevertheless, limited cytotoxicity results from their Cd content (10, 11). In addition, Qdots showed low cytotoxicity when applied to cell culture (12, 13). 

Minimal acute toxicity was found for Qdots *in-vivo*, when rhesus macaques were injected with phospholipid micelle-encapsulated CdSe/CdS/ZnS Qdots. The clearance of Qdots are very slow (10). Qdot-loaded micelles have low acute cytotoxicity when applied *in-vitro* and *in-vivo*. Injection of Qdots into mice tumor bearing nude indicated that they could be used as fluorescent probes for *in-vivo* imaging to study the bio-distribution of nanocarriers and their intracellular pathways. Furthermore, Qdot-loaded micelles were accumulated in the tumor tissue in a passive way (14). In summary, Qdots are precious tools for cellular and molecular imaging techniques to diagnose the nature and stage of cancer and other diseases (15-17).

SSTRs are members of the G-protein coupled receptors (GPCRs) superfamily (18, 19). There are five different subtypes of SSTRs (SSTR_1-5_), SSTR_2_ having been classified into two subtypes, SSTR_2A_ and SSTR_2B_ (20, 21). The blocking of SSTRs with antagonist supresses the interaction of the peptide agonist with SSTRs (22). SSTRs present in numerous normal and diseased cells, are expressed in normal tissues such as the pituitary gland and pancreas (21, 23). SSTRs are also expressed in many tumor cells i.e. small cell lung cancer (24-26), neuroendocrine tumors, and breast cancer (27, 28). 

SST has many functions in mammals such as controling the secretion of growth hormones (29). Moreover, it is widely distributed throughout the central nervous system and peripheral tissues there playing numerous roles (30, 31). Furthermore, SST inhibits the regulation of many endogenous cell functions, including the modulation of neurotransmission, motility, cell proliferation, and cell secretion (32, 33). The limited stability and the presence of many functional groups within SST make the interaction with other compounds such as PEGylation with thiolated-PEG difficult (34-36). Trautʹs Reagent (2-iminothiolane) is a small thiolation compound that reacts with primary amines (e.g., lysine side chains) to add a small spacer arm (8.1 angstroms) terminated by a free sulfhydryl group (—SH). This thiolation is very fast and specify and no other thiolation method can perform like traut´s reagent except thiolation with chemical reaction. Once added, sulfhydryl groups can be specifically targeted for reaction in a variety of useful labeling (37). The main aim of this study is to conjugate SST to quntum dots nanoparticles carrying poly ethylene glycol amine (Qdots-PEG-NH_2_). For this conjugation SST was thiolated then conjuagted to Qdots-PEG-NH_2_-sulfo-SMCC as a step that can facilitate the activation of Qdots-PEG-NH_2_ with SST. The conjugation started with the thiolation of SST using Traut’s reagent. On the other hand, the Qdots-PEG-NH_2_ chains were activated by sulfo-SMCC dissolved in phosphate buffer. The Qdots-PEG-NH_2_-sulfo-SMCC were conjugated to the thiolated-SST to form Qdots-PEG-NH_2_-SST (Qdots-SST). The cellular uptake of Qdots-SST was studied using Triple negative breast cancer cells (HCC-1806). HCC-1806 cells were incubated with Qdots-PEG-NH_2_, Qdots-SST and Qdots-SST in the presence of free SST for 1 h. The number of all different types of Qdots nanoparticles per cell were determined using ICP-OES. 

## Experimental

*Materials*

Somatostatin acetate (SST) was kindly supplied from CuraMED Pharma GmbH (karlsruhe, Germany). Hydrogen tetrachloroaurate tri-hydrate, Traut´s reagent (2-Iminothiolane hydrochloride), Ellman’s reagent (5,5ʹ-dithiobis-(2-nitrobenzoic acid)), Qdots carrying PEG-amine were purchased from invitrogen (Darmstadt, Germany). The ultrafiltration units with a 100-kDa cut-off membrane were purchased from Amicon Ultra-4 Millipore (Billerica, MA). Triple negative breast cancers cells (HCC-1806) were purchased from ATCC Middlesex (TW11 0LY, U.K.). Dulbecco`s phosphate buffered saline (pH 7.4), Dulbeccoʹs Modified Eagle Medium and Leibovitz′s L-15 were purchased from invitrogen, (paisley, UK).

The purified water used for all experiments was obtained using a Milli-Q water purification system from Millipore (Schwalbach, Garmany). All glassware was thoroughly washed with freshly prepared aqua regia (HCl/HNO_3,_ 3:1), extensively rinsed with Millipore water several times and oven-dried at 50 °C for 2-3 h before use.

*Bio-conjugation of somatostatin to Quantum dots PEG amine*

**Figure 1:**
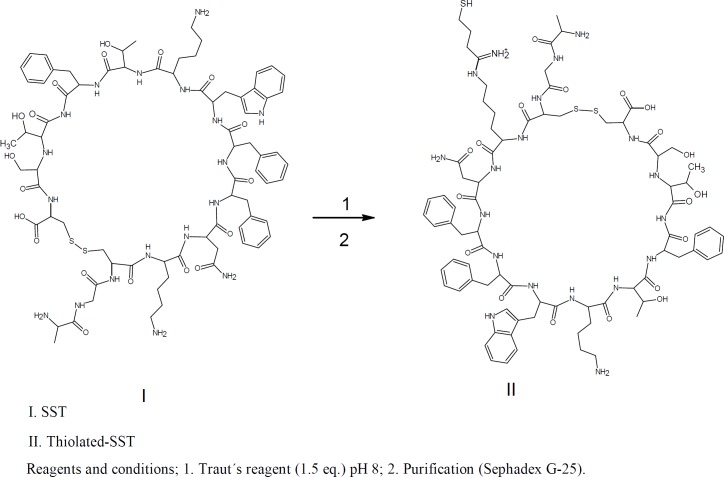
Thiolation of SST with Traut’s reagent. Running through an equilibrated sephadex G-25 mini-column purified the reaction mixture.

**Figure 2:**
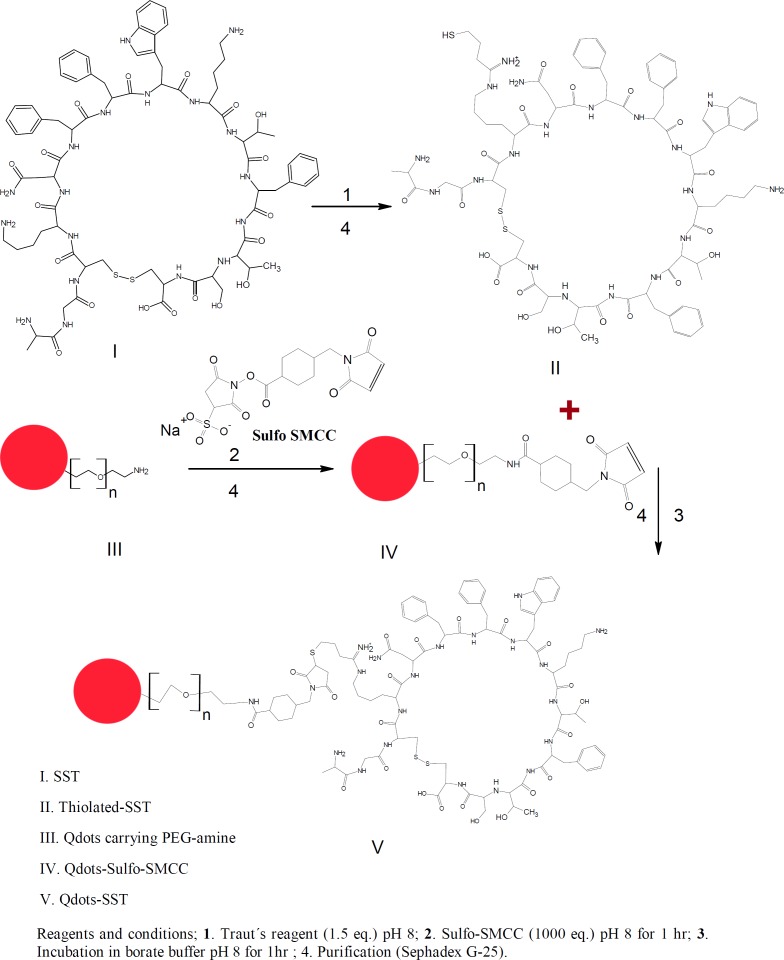
Bio-conjugation of SST to Qdots-PEG-NH2. Qdots-PEG-NH2 activated with sulfo-SMCC. The activated Qdots-PEG-NH2-sulfo-SMCC was conjugated to the thiolated-SST to form Qdots-SST. The obtained bio-conjugate was puriﬁed by using sephadex G-25.

For conjugation of SST to Qdots-PEG-NH_2_, SST was thiolated using Traut’s reagent. Furthermore, the Qdots-PEG-NH_2_ were subsequently activated by a 500-fold molar excess of sulfo-SMCC in phosphate buffer to yield a ﬁnal volume of 250 μL. The activated Qdots-PEG-NH_2_-sulfo-SMCC was conjugated to the thiolated-SST to form Qdots-SST (Figures 1&2). The obtained bio-conjugate was puriﬁed by centrifugation at (5000 rpm for 5 min) using an ultraﬁltration tube (Amicon Ultra-4, 100K MWCO; GE Healthcare). For thiolation of SST with Traut´s reagent, SST was dissolved in phosphate buffer (pH 8, 1 mM EDTA, 0.1 M). Traut´s reagent was dissolved in the triethanolamine buffer (pH 8). The role of EDTA in the sample was to chelate divalent metal ions which can oxidize sulfhydryl groups. Triethanolamine buffer was used to dissociate HCl from Traut´s reagent to give free Traut´s reagent. Typically, 500 µL of 1.2 mM SST was reacted with different equivalents of Traut´s reagent (1, 2, 3, 4 and 5 eq.). The reactants were incubated at room temperature and stirred at 600 rpm for 60 min (38). The reaction mixture was purified by running through an equilibrated sephadex G-25 mini-column. The numbers of sulfhydryl groups were determined using Ellman’s reagent (Figure 2).

*Characterization of the thiolated-SST*

**Figure 3:**
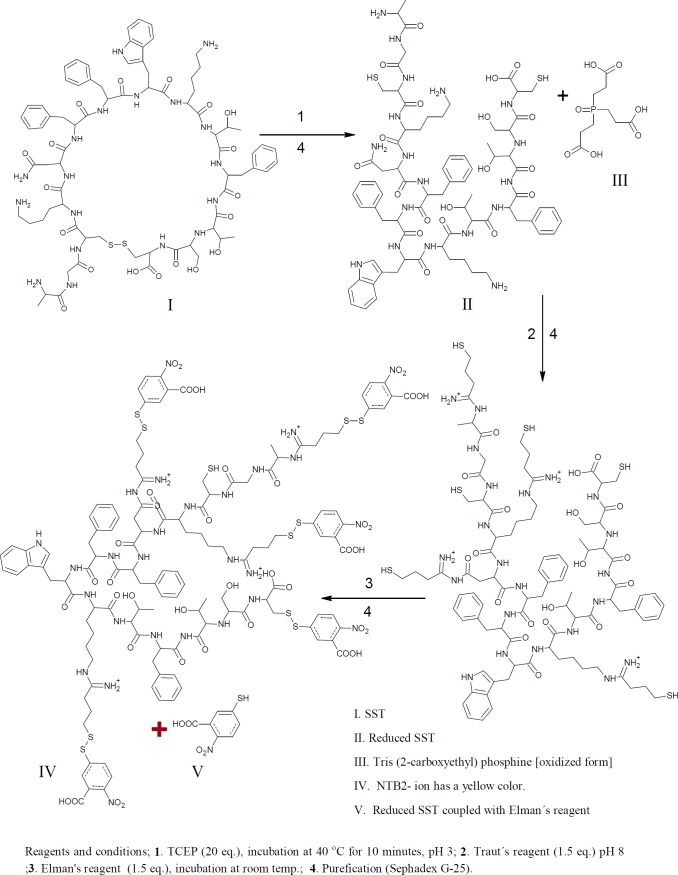
Determination of the number of free sulfhydryl groups. SST was reduced with TCEP and then thiolated with Traut´s reagent.

Free sulfhydryl groups were assayed with Ellman’s reagent (DTNB); 5,5ʹ-Dithiobis-(2-Nitrobenzoic Acid). Thiols react with this compound cleaving the disulfide bond to give 2-nitro-5-thiobenzoate (TNB) as a second product which ionizes to the TNB^2^ dianion in water at neutral and alkaline pH. This TNB^2^ ion has a yellow color. Addition of one mole of thiol releases one mole of TNB. The TNB^2^ is quantified in a spectrophotometer by measuring the absorbance of visible light at 412 nm (Figures 1&3) (39-42).

*Quantitating the sulfhydryl groups of thiolated-SST*

A set of tubes was used, each containing 50 µL of Ellman’s reagent plus 2.5 mL of reaction buffer (Table 1). A 250 µL of each standard or unknown sample was added to test tubes prepared beforehand. For the unknown samples, dilutions have done so that the 250 µL samples were used to assay reaction which has a sulfhydryl concentration in the working range of the standard curve (0.1 - 1.0 mM is ideal) (Table 1). The content of each tube was mixed and incubated at room temperature for 15 min and absorbance was measured at 412 nm. The concentration of sulfhydryls in the sample was calculated from the molar extinction coefficient of TNB. The most accurate results were obtained from the linear portion of the standard curve. Concentrations exceeding 1 mM free sulfhydryl will result in high absorbance values and so less accurate estimation of the concentration based on the extinction coefficient of TNB.

*Determination the number of free sulfhydryl groups of reduced SST*

tris(2-carboxyethyl)phosphine hydrochloride, TCEP is non-volatile, odorless, and unlike most other reducing agents, resistant to air oxidation. TCEP is very stable, more effective, and able to reduce disulfide bonds at lower pH. The aim of this reaction is to confirm the thiolation of SST with Traut´s reagent, because the thiolated products react with Ellman’s reagent, and the numbers of thiol groups will be increased (Figure 1). Typically, 1.2 mL of TCEP 20.4 mM concentration mixed with 0.8 mL of SST (50 µM). The reactants were stirred together at 600 rpm at room temperature for 60 mins. For complete reduction, peptide and reducing agent were stirred again at 40 °C for 10min. The disulfide bond of SST was reduced using 20 folds molar excess of TCEP. For dissolving TCEP, TCEP was dissolved in 0.17 M potassium citrate buffer. The reduced SST was thiolated with Traut´s reagent as previously proceeded. Thiolated reduced SST was reacted with Ellman’s reagent (43-45). The product was purified by running through an equilibrated Sephadex G-25 mini-column. 

**Table 1:** Different concentrations of cysteine standards.

**Standard**	**Volume of Reaction** **Buffer**	**Amount of Cysteine** **(M.W. = 175.6)**	**Final Concentration**
A	100 mL	26.34 mg	1.5 mM
B	5 mL	25 mL of Standard A	1.25 mM
C	10 mL	20 mL of Standard A	1.0 mM
D	15 mL	15 mL of Standard A	0.75 mM
E	20 mL	10 mL of Standard A	0.5 mM
F	25 mL	5 mL of Standard A	0.25 mM
G	30 mL	0 mL	0.0 mM (Blank)

*HPLC identification of thiolated-SST labeled with eosin-5-maleimide*

**Figure 4:**
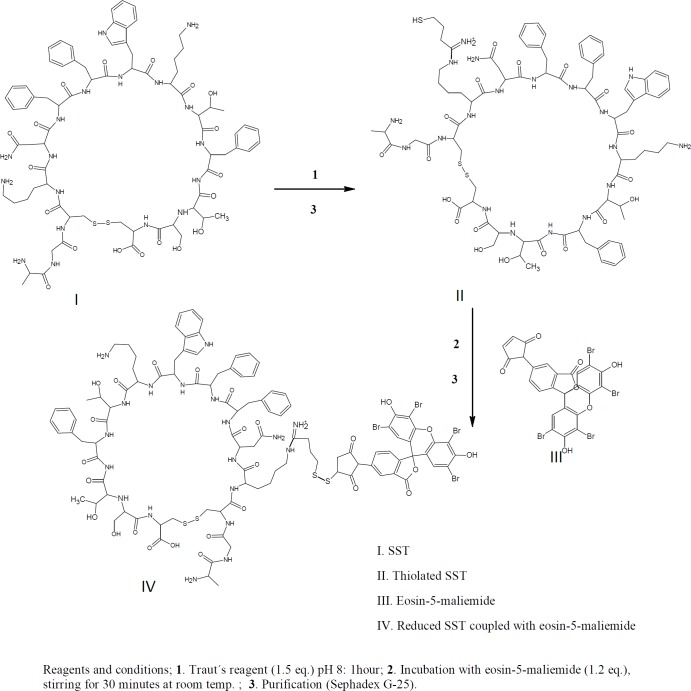
Identification of thiol groups of thiolated-SST. The thiolated-SST labeled with eosin-5-maliemide. Fluorescence detection detected the peaks at wavelengths of 524 nm / 545 nm.

For confirmation of thiolation of SST with Traut´s reagent, eosin-5-maleimide was used for labeling of the thiolated-SST. Eosin-5-maleimide can be used as fluorescent probes or as photosensitizers and can be detected using fluorescence detector at wavelengths of 524 nm/ 545 nm (46, 47). In brief, 1 mL of 1.2 mM SST was reacted with (1.5 eq) Traut’s reagent. The products were purified by running in Sephadex G-25 mini-column as shown in Figure 4. 

Then, thiolated-SST labeled with (1.5 eq) eosin-5-maleimide. Eosin-5-maleimide solution was mixed with thiolated-SST, the reactants were stirred at 600rpm for 30 min. The products were purified by running in Sephadex G-25 mini-column, and identified by thin layer chromatography. HPLC analysis was performed for SST using a linear gradient from 26% to 39% acetonitrile in water, with 0.1% TFA as mobile phase at a flow rate of 1.0 mL/min using a C18-reversed phase analytical column. Fluorescence detection showed the peaks at wavelengths of 524 nm / 545 nm.

*Cellular uptake study*

To study the cellular uptake of Qdots-SST, Triple negative breast cancers cells (HCC-1806) were chosen as a model cell line. HCC-1806 cells were incubated with unmodified Qdots-PEG-NH_2_ (as control nanoparticles) and Qdots-SST for 1 h in RPMI 1640 medium containing serum. The number of nanparticles per cell was determined by inductively coupled plasma optical emission spectroscopy, ICP-OES. The initial concentration of Qdots-PEG-NH_2_ in the culture medium was 20 nM for unmodified Qdots-PEG-NH_2_ and Qdots-SST.

## Results and Discussion

*Derivatization of SST with Traut’s reagent, and quantitating the number of sulfhydryl groups using Ellman´s reagent*

**Figure 5:**
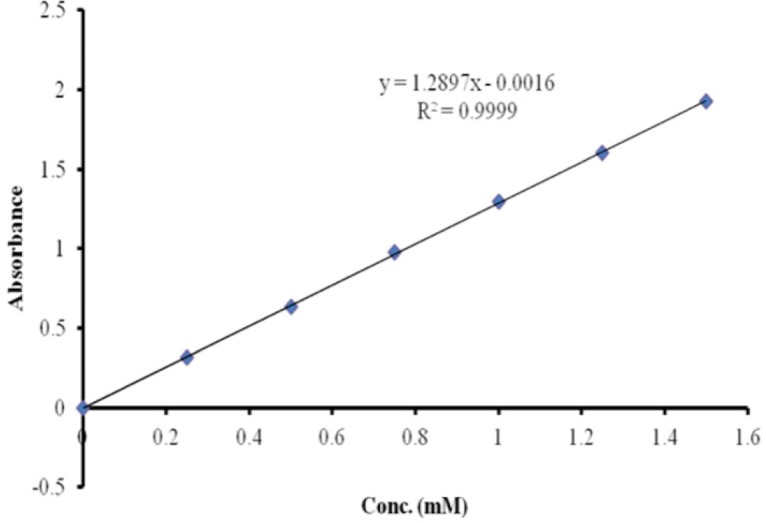
Calibration curve of cysteine for estimation of sulfhydryle groups using Ellman´s reagent. The calibration curve of cysteine was linear from 0 mM to 1.5 mM.

SST was derivatized using Traut´s reagent to form thiolated-SST (48). The conditions used for this reaction were applied to SST and SST analogue (seglitide), which contained two lysine and one lysine residue respectively. The molar ratio of Traut’s reagent to SST and seglitide was 10:1, and the reaction was carried out at room temperature. To determine the number of sulfhydride groups, Ellman’s reagent method was used. The calibration curve of cysteine was used to estimate the number of sulfhydride groups (Figure 5). The calibration curve of cysteine was linear from 0 mM to 1.5 mM. To examine the linearity of the calibration curve, 7 different concentrations of cysteine solutions were measured (0, 0.25, 0.5, 0.75, 1, 1.25 and 1.5 mMol/L). Regression analysis gave a linear relationship: y = 1.3 x - 0.0016 mol/L, (R² = 0.9999) (49). 

**Figure 6:**
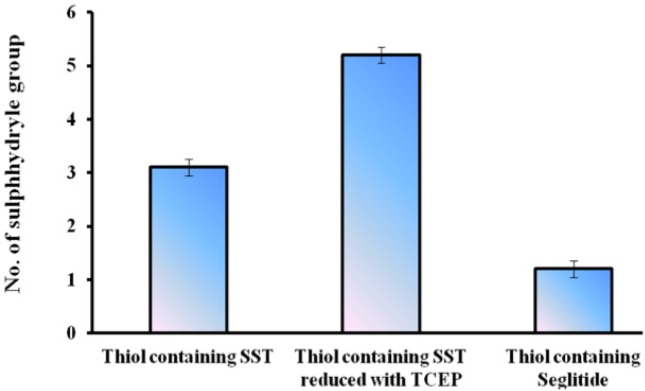
Quantitating the sulfhydryl groups of SST, SST reduced with TCEP and seglitide using a cysteine calibration.

The numbers of free sulfhydryl groups of thiolated-SST were 3.2 (3 thiol groups ), the numbers of free sulfhydryl groups of thiolated-SST reduced by TCEP were 5.6 (6 thiol groups) (Figure 6). This is due to SST contains four free amino groups and two SH groups after reduction with TCEP. The higher number of thiol groups may be due to the breakage of the intrinsic disulfide bond in the molecule, while the number of free sulfhydryl groups of thiolated-seglitide was just only one (38, 48, 50). To confirm the thiolation of SST, SST was reduced with TCEP. increase in number of sulfhydryl groups in reduced-thiolated-SST confimed that the number of sulfhydryl groups in thiolated-SST were 3. The derivatization of SST with Traut´s reagent is very simple and versatile. The number of sulfhydryl groups can be controlled by the molar ratio of Traut´s reagent to SST. The thiolation of seglitide and reduction of SST with TCEP were proceeded to confirm the thiolation of SST.

*HPLC analysis of thiolated-SST*

**Figure 7:**
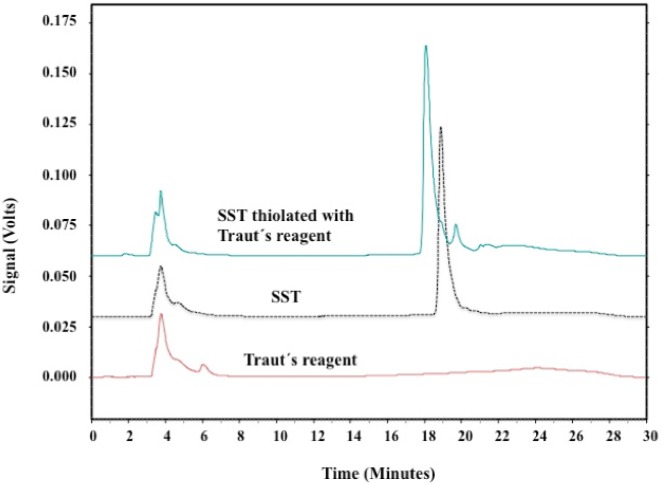
HPLC chromatographs of SST reacted with Traut´s reagent. Signals were detected at 274 nm. Single peak eluted at about 19 min corresponded to SST. Thiolated-SST eluted at 18.6 min.

The thiolation of SST was investigated using HPLC. Figure 7 shows the HPLC-chromatograph for SST before and after thiolation with Traut’s reagent. The single peak eluted at about 19 min corresponded to SST. Thiolated-SST eluted at 18.6 min. The signal consists of multiple neighbouring peaks, these neighbouring peaks appeared between 20.5 and 23 min (Figure 7) and could be related to SST in isomerized form. Neither thiolated-SST nor SST degradation products were detected. Thiolated-SST eluted earlier than SST which is related to the increase in hydrophilicity. Traut´s reagent showed no significant signal, and do not interfere the analysis of the peptides.

**Figure 8:**
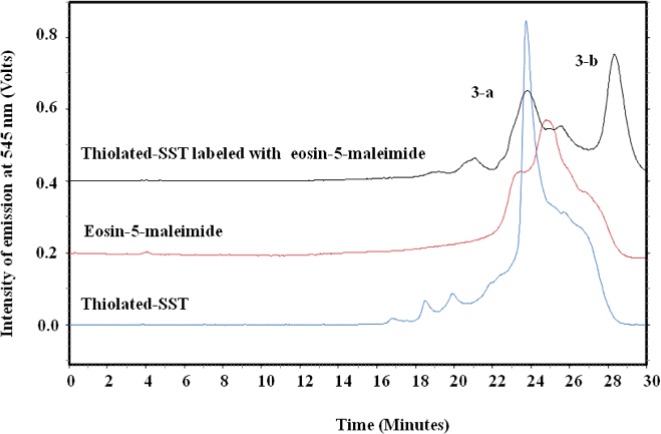
HPLC chromatograph of SST, SST thiolated with Traut´s reagent and labeled with eosin-5-maliemide. Peak (3-a) corresponds to thiolated-SST. Peak (3-b) represents the thiolated-SST labeled with eosin-5-maleimide, which shows a conversion of about 59.1%, confirming that the SST was thiolated by Traut´s reagent. The thiol-reactive eosin-5-maleimide signals were detected using fluorescence detection at 545nm.

To confirm the thiolation of SST with Traut´s reagent, eosin-5-maleimide was conjugated to the sulfhydryl group of thiolated-SST. The thiol-reactive eosin-5-maleimide can be detected using fluorescence detection at 545 nm (46, 47). Figure 8 shows the chromatograph of the reaction mixture of thiolated-SST labeled with eosin-5-maleimide. Peak (3-a) corresponds to thiolated-SST. Its intensity decreased from 0.85 to 0.22 during the reaction, which indicates that 26% did not interact with eosin-5-maleimide and remained in thiolated form. Furthermore, HPLC analysis showed that the peak of eosin-5-maleimide showed some impurities as reported by the supplier. The chromatogram of thiolated-SST showed some impurities from Traut´s reagent that could not be separated. Peak (3-b) represents the thiolated-SST labeled with eosin-5-maleimide, which shows a conversion of about 59%, confirming that the SST was thiolated by Traut´s reagent. 

For the conjugation of thiolated-SST to Qdots-PEG-NH_2_, Qdots-PEG-NH_2_ were rendered thiol-reactive upon treatment with sulfo-SMCC, subsequently reacted with thiolated-SST and purified by gel filtration chromatography as shown in Figure 2. The thiolated-SST reacted in a molar ratio of 20:1 with Qdots-PEG-NH_2_ activated with sulfo-SMCC calculated based on the number of activated PEG chains. The produced nanoparticles were incubated with HCC1806 in culture media to study the internalization of Qdots-SST.

*Cellular uptake study*

**Figure 9:**
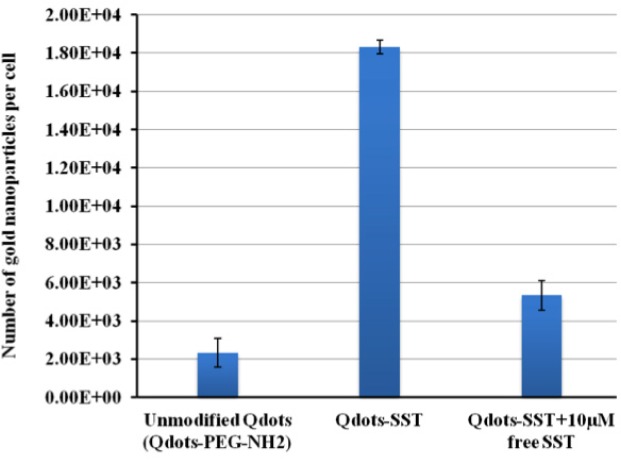
The bio-distribution of Qdots-PEG-NH2 in HCC1806 cells from mice as determined by ICP-OES.

Qdots-SST were internalized in higher amounts (estimated number, 18333 ± 356 per cell) more than the unmodified particles (estimated number 2339 ± 755). The internalization of Qdots-SST was suppressed in the presence of high concentration of SST, the concentration used was 10 µM and the estimated number was 5339 ± 758 (Figure 9). However, the higher amounts of SST up to 10 µM displaced the nanoparticles from the receptor. Hence, it can be concluded that the difference in the cellular uptake of Qdots-SST is due to differences in surface properties and not in the size of Qdots-PEG-NH_2_. Nanoparticle interaction with cells is an issue of importance for targeting of these particles to different cells. Furthermore, this is an active targeting to SSTRs with also counteract unwanted interactions with cell receotor such as the non-specific enternalizarion. SST as SSTRs agonist could easily fit on the SSTRs which increase the internalization of Qdots-SST, whilest blocking of these receptors by high concentration of SST led to decrease of the internalization of Qdots-SST.

The thiolated-SST was reacted with Ellman’s reagent for determination of the number of sulfhydryl groups. The disulfide bond of Ellman´s reagent cleaves to give TNB as a second product which ionized to TNB^2^-dianion in water at neutral and alkaline pH. This TNB^2^ ion has a yellow color. The TNB^2^ is quantified in a spectrophotometer by measuring the absorbance at 412 nm.

## Conclusion

Thiolation was necessary for the conjugation of SST to Qdots-PEG-NH_2_. The results confirm that the SST was sufficiently thiolated by Traut’s reagent. Ellman’s reagent was used to determine the number of sulfhydryle groups. In addition, higher amounts of Qdots-SST particles internalized per cell in HCC-1806 cell lines compared to unmodified Qdots-PEG-NH_2_ and Qdots-SST in the presence of high conc of free SST. Such internalization depends on the surface properties of the cells not on the size of particles as shown when the receptors were blocked by incorporation of free agonist peptide. Finally, the principle has been proofed, and we will focus in the future to deliver these Qdots-SST to different tumor cells.
